# Molecular Epidemiology and Antibiotic Susceptibility of Livestock *Brucella melitensis* Isolates from Naryn Oblast, Kyrgyzstan

**DOI:** 10.1371/journal.pntd.0002047

**Published:** 2013-02-28

**Authors:** Joldoshbek Kasymbekov, Joldoshbek Imanseitov, Marie Ballif, Nadia Schürch, Sandra Paniga, Paola Pilo, Mauro Tonolla, Cinzia Benagli, Kulyash Akylbekova, Zarima Jumakanova, Esther Schelling, Jakob Zinsstag

**Affiliations:** 1 State Veterinary Department, Bishkek, Kyrgyzstan; 2 Swiss Tropical and Public Health Institute, Basel, Switzerland; 3 University of Basel, Basel, Switzerland; 4 Naryn Zonal State Centre for Veterinary Diagnostic, Naryn, Kyrgyzstan; 5 Labor Spiez, Spiez, Switzerland; 6 Institute of Veterinary Bacteriology, Vetsuisse Faculty of the University of Berne, Berne, Switzerland; 7 Cantonal Institute of Microbiology, Bellinzona, Switzerland; 8 Republican State Centre for Veterinary Diagnostic, Bishkek, Kyrgyzstan; Institut Pasteur, France

## Abstract

The incidence of human brucellosis in Kyrgyzstan has been increasing in the last years and was identified as a priority disease needing most urgent control measures in the livestock population. The latest species identification of *Brucella* isolates in Kyrgyzstan was carried out in the 1960s and investigated the circulation of *Brucella abortus, B. melitensis, B. ovis,* and *B. suis.* However, supporting data and documentation of that experience are lacking. Therefore, typing of *Brucella spp.* and identification of the most important host species are necessary for the understanding of the main transmission routes and to adopt an effective brucellosis control policy in Kyrgyzstan. Overall, 17 *B. melitensis* strains from aborted fetuses of sheep and cattle isolated in the province of Naryn were studied. All strains were susceptible to trimethoprim-sulfamethoxazole, gentamicin, rifampin, ofloxacin, streptomycin, doxycycline, and ciprofloxacin. Multilocus variable number tandem repeat analysis showed low genetic diversity. Kyrgyz strains seem to be genetically associated with the Eastern Mediterranean group of the *Brucella* global phylogeny. We identified and confirmed transmission of *B. melitensis* to cattle and a close genetic relationship between *B. melitensis* strains isolated from sheep sharing the same pasture.

## Author Summary

Brucellosis is a bacterial disease causing abortion in cattle, sheep, and goats. It is transmissible to humans by direct transmission and the consumption of untreated milk. Brucellosis has become more and more frequent in Kyrgyzstan in the last decades, and its control has been made a priority. Knowing the bacterial strain circulating is important for the understanding of the transmission and the selection of interventions. The latest identification of *Brucella* in Kyrgyzstan dates from the 1960s. We report the molecular characterization 17 strains identified as *Brucella melitensis* from Naryn oblast. Strains were mainly isolated from sheep but also from cattle. All strains were susceptible to a series of antibiotics. We hence identified and confirmed transmission of *B. melitensis* among sheep which is likely the most important host species. We found close genetic relationship between *B. melitensis* strains isolated from cattle sharing the same pasture with sheep. Our results support the strategy of pursuing a mass vaccination of livestock in Kyrgyzstan. Further research is needed to identify the most important circulating strains in humans.

## Introduction

Agriculture is a key component of Kyrgyzstan's economy and livestock play a major role in the daily lives of the population. Sixty four percents of the population live in rural areas and rely on agriculture for their livelihoods. Up to 76% of the rural population of the country is classified as poor. [1].

Since independence in 1991, veterinary support ceased then largely and the incidence of diseases transmitted from animals to humans (zoonoses) has increased dramatically in many regions in Kyrgyzstan. Brucellosis, anthrax, rabies and echinococcosis are public health concerns and constitute a serious risk to the human and the livestock health. The incidence of brucellosis has increased steadily and Kyrgyzstan has now one of the highest human brucellosis incidences worldwide (annual incidence: 77.5 new cases per 100,000 people in 2007) [2].

Currently, Kyrgyz communities are concerned about the effective reduction of the brucellosis burden in people and livestock.

The latest species identification of *Brucella* spp. cultures in Kyrgyzstan was done in the 1960ies. Both *B. abortus* and *B. melitensis* were isolated from cattle. *B. melitensis* infections in cattle were thought to be a spill-over from sheep. Smirnov and Tretyakova noted that abortions in cows after immunization with S19 were most often seen in herds that were infected with *Brucella* spp. *B. melitensis* was isolated from vaccinated and non-vaccinated sheep [3], [4]. the authors concluded that B. melitentsis steadily adapted to sheep [5].

At present, the circulating genotypes of *Brucella* spp. are not known. This is true for virtually all Central Asian regions. Bacteriological confirmation of *Brucella* spp.-induced abortions is almost absent, because owners do not report suspected abortions to the veterinary services. Here we report recently isolated *Brucella* spp. strains from sheep and cattle, which were collected in addition to a representative national study on brucellosis sero-prevalence in humans and livestock [6]. and to cost of disease studies in Kyrgyzstan (data not shown). The results contribute to the understanding of the main transmission routes and effectively inform brucellosis control policy in Kyrgyzstan.

## Materials and Methods

### Sampling sites and survey

The study was performed in the province of Naryn oblast, which has the highest human brucellosis incidence in Kyrgyzstan and most of its population has an income through selling of animals and animal products. First primary isolations of *Brucella* strains from aborted fetuses were done at the veterinary laboratory of the Naryn province in November 2008. All public and private veterinarians were informed about the ongoing project on brucellosis. Farmers were informed beforehand and asked to report abortions through local village veterinarians; leaflets with information were distributed through veterinarians and announcement was published in the province newspaper. Abortions from sheep and cattle were collected during the lambing seasons of 2009 and 2010. In general, the lambing season starts in January and continues until March and April and thus first abortions can occur in late November/December. Veterinarians brought the collected specimens – aborted sheep and cattle fetuses - dissected on site - to the Naryn laboratory. Stomach content was collected in tubes and liver, spleen, kidney, lung, heart and other tissues were collected in plastic bags. Veterinarians collected accompanying basic information on the animals and farms such as geographic position and keeping of other than affected animals. Two weeks after the abortion, a visit to the affected farm allowed blood sampling of farm animals for serology (data not shown) and an interview with the livestock holders to obtain epidemiological data with a questionnaire. Total number of fetuses collected by the veterinarians was 125 from whole district and positive isolates by the Urease and Oxidase were selected for further study.

### Cultures

Primary cultures were done at the Naryn zonal Center for Veterinary Diagnostic and specimens were frozen. When culture was negative, frozen specimens were re-cultured at the Republican Center for Veterinary Diagnostic in Bishkek.

Stomach content and organs of the aborted fetuses were cultured onto Brucella selective agar (bioMérieux, Switzerland) and onto own produced Brucella selective agar (with agar, horse serum and antibiotics from Oxoid, Switzerland). Strains were cultured on Brucella agar at 37°C with 10% CO_2_ for 2 days [7].

### Antibiotic resistance testing

For the investigation of the sensitivity of the cultures to, phenotypic antibiotic resistance to 7 different drugs was assessed by the standard E-tests (bioMérieux, Switzerland) on Mueller-Hinton blood agar (MHS2, bioMérieux SA, France) and their minimum inhibitory concentrations (MIC) were determined additionally. The following antibiotics were tested: trimethoprim-sulfamethoxazole (SXT) (1.25+23.75 µg), gentamicin (GM) (10 µg), rifampicin (RA) (30 µg), ofloxacin (OFX) (1 µg), streptomycin (S), (15 µg), doxycyclin (D), (30 µg), and ciprofloxacin (CIP). (5 µg), Inducible clindamycin resistance test (“D-zone” test) was also carried out for all isolates. Results were interpreted according to the Clinical and Laboratory Standards Institute (CLSI) guidelines; for the purpose of this study, intermediate results were classified as resistant.

### DNA extraction and genotyping

DNA was extracted from one loopful of bacterial cells grown for 48 h on chocolate agar, and single colonies were isolated by using the tissue protocol of the QIAamp DNA minikit (Qiagen, Germany). DNA concentrations were measured by UV spectrophotometry (Shimadzu, Japan). Multiple Loci Variable Number of Tandem Repeat Analysis (16 locus MLVA) typing was performed with the 17 isolates according to the protocol initially proposed by Le Flèche et al. [8]. and modified by Al Dahouk [9]. to include 1 additional locus, bruce19. The protocols are available online on the MLVA-NET for Brucella (http://mlva.u-psud.fr). In brief, the assay comprised the typing of eight mini-satellites of the so-called panel 1 (bruce06, bruce08, bruce11, bruce12, bruce42, bruce43, bruce45, and bruce55), three micro-satellites of the panel 2A (bruce18, bruce19, and bruce21), and five micro-satellites of the panel 2B (bruce04, bruce07, bruce09, bruce16, and bruce30).

The 16 published VNTR loci were PCR-amplified in parallel and the numbers of tandem repeats determined after electrophoresis on agarose gel. DNA extracts of *B. melitensis* 16M^T^ and vaccine strain Rev1 were used as positive controls. The obtained MLVA patterns of each sample were then matched with an online database (http://minisatellites.u-psud.fr/MLVAnet/querypub1.php) for identification.

### MALDI-TOF MS analysis

A small amount of a colony of each pure culture was transferred to a FlexiMass target well using a disposable loop and overlaid with 1.0 µl alpha-cyano matrix solution (CHCA; 40 mg alpha-cyano in 33% acetonitrile, 33% ethanol, 33% ddH2O and 1% trifluoroacetic acid). The spotted solution was air-dehydrated during 1–2 min at room temperature and analysed with MALDI-TOF MS Axima Confidence spectrometer (Shimadzu-Biotech Corp., Kyoto, Japan). The reference strain *Escherichia coli* K12 (GM48 genotype) was used as a standard for calibration and as reference measurement for quality control. Mass spectrometry (MS) analyses were performed in positive linear mode in the range of 2,000–20,000 mass-to-charge ratio (m/z) with delayed, positive ion extraction (delay time: 104 ns with a scale factor of 800) and an acceleration voltage of 20 kV. For each sample, 2×50 averaged profile spectra were stored and used for analysis. All spectra were processed by the MALDI MS Launchpad 2.8 software (Shimadzu Biotech) with baseline correction, peak filtering and smoothing. A minimum of 20 laser shots per sample were used to generate each ion spectrum. For each bacterial sample, 50 protein mass fingerprints were averaged and processed. Spectra were analyzed using SARAMIS (Spectral Archive and Microbial Identification System, AnagnosTec GmbH) at default settings. Cladistic analysis were based on the peak patterns of all analyzed strains submitted to single-link clustering analysis using SARAMIS with an error of 0.08% and a m/z range of 2,000 to 20,000 Daltons.

### Data analysis

Allelic diversity was calculated using the formula below, where *x_i_* is the
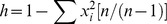
relative frequency of the *i*th allele at the locus, *n* the number of isolates in the sample and *(n/(n-1)* is a correction for bias in small samples [10]. VNTR data was the basis for the phylogenetic analysis using SAS (Statistical Analysis Systems Inc. Cary, USA) proc cluster using the unweighted pair-group method with arithmetic averages, (UPGMA). For the assessment of the phylogenetic place of the Kyrgyz isolates, strains were selected from the online database by Maquart [11], [12]. (1471-2180-9-145-S1.xls; http://www.biomedcentral.com/1471-2180/9/145). Isolates were selected to reflect the diversity of geographical origin and the different biovars. Phylogenetic trees were drawn using SAS proc tree.

### Ethics statement

The Ethics Committee of the University and the state of Basel has approved this study without restrictions in the meeting of January 11, 2007 (Reference number 02/07). The project conforms with the ethics requirements on animal testing (Published in Schweiz. Ärztezeitung, 2006, Band 87, S. 832–837) by the Swiss Academy of Medical Sciences and the Swiss Academy of Natural Sciences. Animal owners were asked for consent to test aborted fetuses of their livestock for brucellosis.

## Results

**Figure 1:**
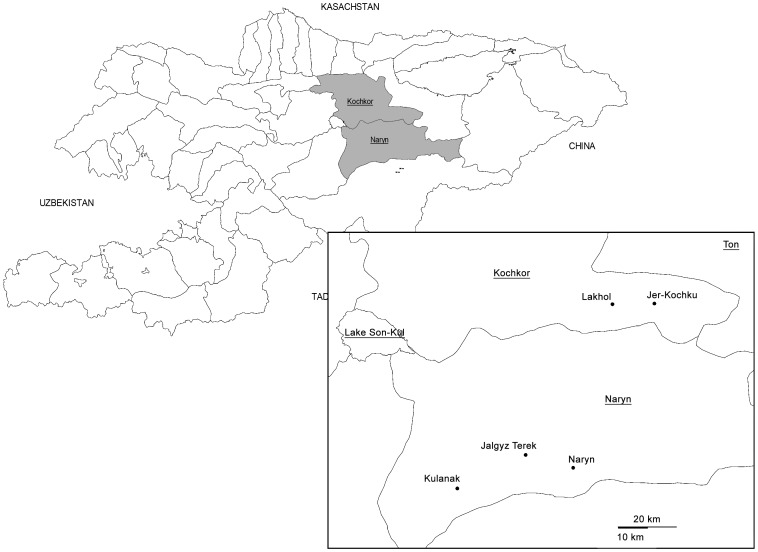
Geographic location of the Naryn oblast and the villages from were *Brucella melitensis* was isolated.

**Figure 2:**
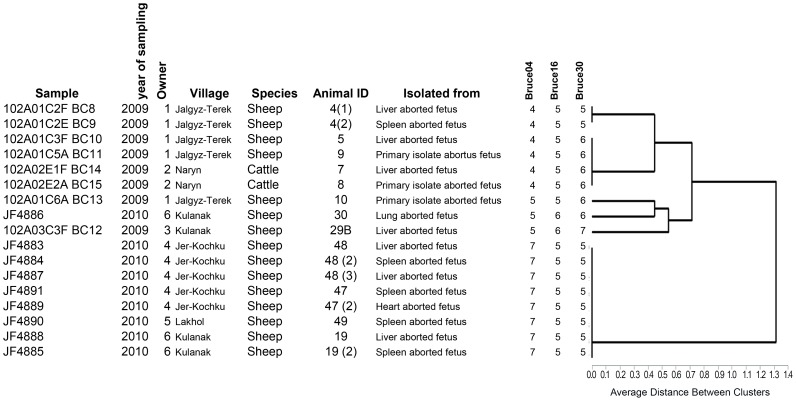
Dendrogram showing the relationship of the 15 sheep and two cattle isolates of *Brucella melitensis*.

**Figure 3:**
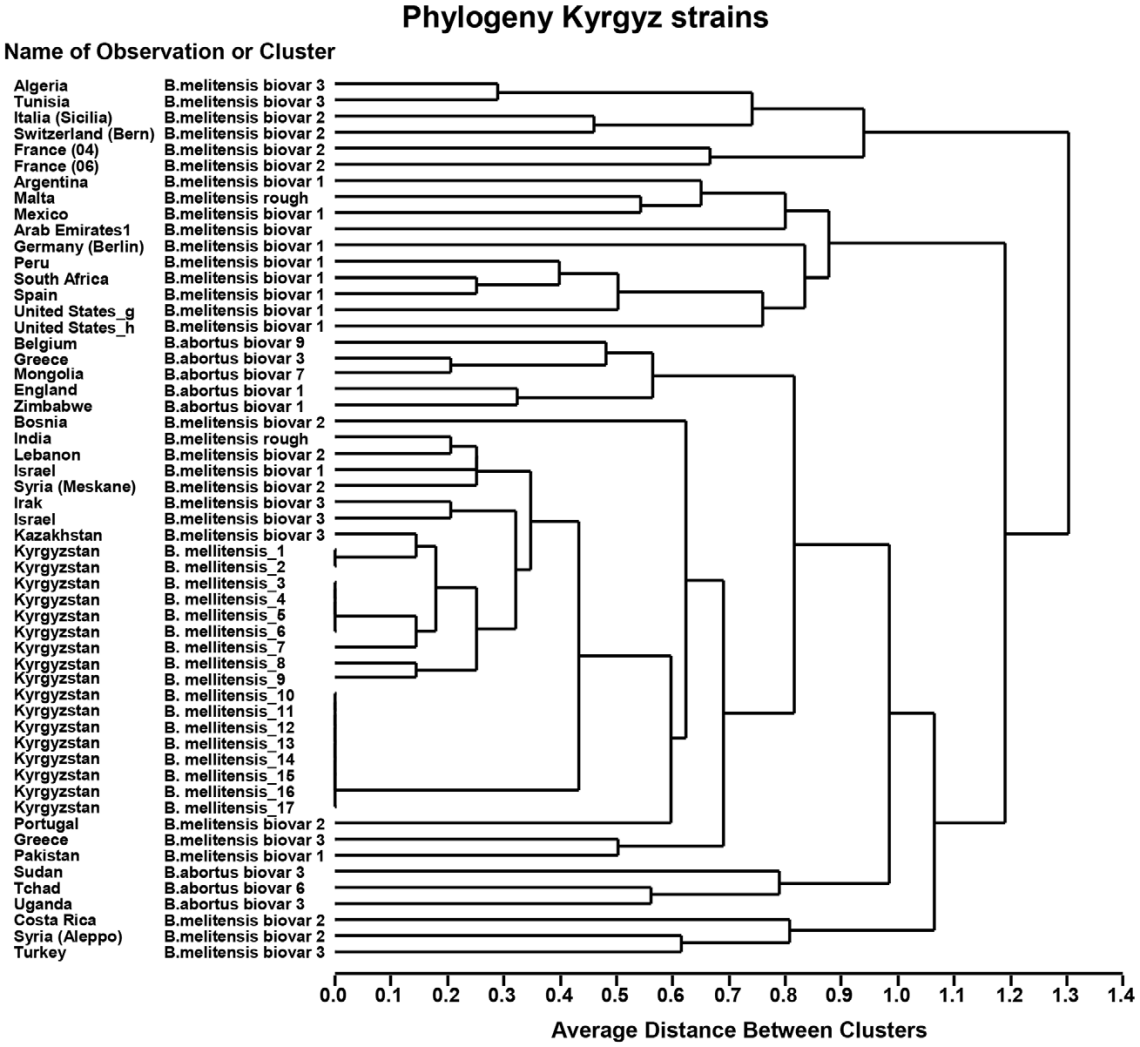
Dendrogram the relationship of the Kyrgyz isolates when compared to a global database 11.

Livestock systems and management of herds from which *B. melitensis* were isolated varied between owners. Livestock owners kept cattle and small ruminants together and practiced seasonal transhumance to high-altitude pastures. They sometimes also kept entrusted animals from several owners and traded actively animals. During the lambing seasons 2009 and 2010 in Naryn, 125 aborted fetuses (112 from sheep and 13 from cattle) were collected in the 4 villages and the city of Naryn (Figure 1). The rate of isolation for sheep was 8.9% and for cattle is 15% but the difference is not statistically significant. Urease and oxidase positive cultures were selected and 17 out of 23 isolates were confirmed *B. melitensis* by MALDI-ToF MS and MLVA-16 (Figure 2). The dendrogram is based on the MLVA-16 genotyping assay showing the relationship of the 15 sheep and two cattle isolates of Brucella melitensis. For each locus showing variability, the number of tandem repeats is presented. Additional information is provided on the type of sample, the local strain designation, the serial number of the animal owner and the name of the village in Naryn oblast Numbers in brackets indicate repeated isolates from the same animal. Isolates not indicated as primary were frozen prior to cultivation. Of the 17 isolates, 15 were isolated from sheep and two from cattle. All strains were susceptible to the tested antibiotics. The allelic diversity of VNTR (*h*) was low, with only three loci showing variation in the numbers of repeats. For locus 4 it was 0.6, for locus 16 0.16 and 0.49 for locus 30 (Table 1). All other loci did not show any variation. Eight out of 17 strains grouped into 6 different clusters. However, it has to be noted that more than one isolate was obtained from four animals. Isolates of cluster 2 were found in herds of two different owners in sheep and cattle. With regard to the geographical location, the Kyrgyz isolates are closest to strains from Kazakhstan, Israel and Iraq which are all biovar3 (Figure 3) [11].

**Table 1:** Allelic diversity of VNTR loci (all other loci where equal).

reference	Number of repeats				allelic diversities
# of copies	4	5	6	7	
bruce 4	0,35/6	0,18/3		0,47/8	0,601
bruce 16		0,88/15	0,12/2		0,162
bruce 30		0,59/10	0,35/6	0,06/1	0,496

Nominator – allelic diversity index.

Denominator – number of repeats.

## Discussion

*B. melitensis* isolates from Kyrgyzstan appear to be close to the so-called Eastern Mediterranean group (Figure 3) [11], but a more detailed analysis and more isolates are required to conclusively determine the position of Kyrgyz Brucella in the global phylogeny. All *B. melitensis* isolates from Naryn Oblast were closely related according to VNTR patterns. Isolates belonging to second cluster from the top (Strain No. 3–6) (Figure 2) were found in the herd of two owners of sheep and cattle, indicating that strains circulated between farms and were transmitted between small ruminants and likely to cattle during communal grazing. These two owners live 45–50 km apart. The owner of the cattle lives in the city of Naryn and his cattle graze on a summer pasture with several other animals suggesting rural/urban spill over through sharing of common pasture. The 8 isolates (sixth cluster from the Top in Figure 2) from sheep stem from Jer-Kochku and Lakhol, two villages 10 km apart. The animals from which they originated use the same pasture for grazing, except for the two strains from Kulanak which is located at more than 80 km from Jer-Kochku and Lakhol. This may indicate a contact relationship between Kulanak, Jer-kochku and Lakhol (Figure 1). Owner 1 had sheep in which three *B. melitensis* genotypes are present. A better understanding of the contact network of each animal owner could possibly further explain genetic diversity.

Multiple strains were isolated from liver, spleen and heart in three animals (Figure 2). Isolates from different organs of the same animal had always the same VNTR pattern, hinting to a likely monoinfection. The isolation of *B. melitensis* in sheep and cattle is the first recent confirmation by culture since the 1960ies in Kyrgyzstan. It was expected because brucellosis in cattle was not a problem a decade ago and increasing sero-prevalences and brucellosis abortions in cattle were observed during the past years. It was therefore speculated that cattle may be a spill-over host of *B. melitensis* from small ruminants. More isolates are needed to further consolidate this finding. If confirmed, this may have policy implications for ongoing pilot mass livestock vaccination campaigns, considering cattle vaccination. We found no antibiotic resistance and therefore the standard regimen used in Kyrgyzstan (i.e., Gentamicin plus Doxicycline) is likely to be adequate for humans. However, human isolates should be tested as well. The use of antibiotics in livestock is clearly not recommended.

This study confirms ongoing transmission of *B. melitensis* in sheep and likely to cattle in the province of Naryn in Kyrgyzstan. The high genetic homogeneity indicates rather clonal expansion and ongoing transmission, confirming serological observations [6]. The role of cattle in the transmission of *B. melitensis* transmission should be examined more closely. Further studies on human brucellosis strain characteristics are needed to confirm sheep as the suspected principal source of livestock to human transmission [6]. For this purpose more discriminatory methods than VNTR may be needed. Further collection of isolates from aborted fetuses including information on contact networks are needed to monitor the success of the ongoing mass vaccination campaign and to allow calibrating VNTR dynamics in space and time.

## Conclusion

We conclude that *B. melitensis* is endemic in Naryn oblast and sheep are apparently the main host. *B. melitensis* is also transmitted to cattle. In the study period we observed no abortions in goats and hence consider them less important for brucellosis transmission in Naryn oblast. Our findings confirm an earlier serological study, which related human brucellosis sero-prevalence to sheep but not to goat and cattle [6].
